# Barriers and enablers to diabetic retinopathy screening attendance: Protocol for a systematic review

**DOI:** 10.1186/s13643-016-0309-2

**Published:** 2016-08-11

**Authors:** Ella Graham-Rowe, Fabiana Lorencatto, John G. Lawrenson, Jennifer Burr, Jeremy M. Grimshaw, Noah M. Ivers, Tunde Peto, Catey Bunce, Jill J. Francis, For the WIDeR-EyeS Project team

**Affiliations:** 1 School of Health Sciences, City University London, London, UK; 2 School of Medicine, University of St Andrews, Fife, UK; 3 Clinical Epidemiology Program, Ottawa Hospital Research Institute, Ottawa, Canada and Department of Medicine, University of Ottawa, Ottawa, Canada; 4 Women’s College Hospital - University of Toronto, Toronto, Canada; 5 NIHR Biomedical Research Centre at Moorfields Eye Hospital NHS Foundation Trust and UCL Institute of Ophthalmology, London, UK; 6 NIHR Moorfields Biomedical Research Centre, Moorfields Eye Hospital London, London, UK

**Keywords:** Diabetic retinopathy, Screening, Attendance, Barriers, Enablers, Facilitators, Theoretical Domains Framework, Consolidated Framework for Implementation Research, Thematic analysis

## Abstract

**Background:**

Diabetic retinopathy is a serious complication of diabetes which, if left untreated, can result in blindness. Population screening among people with diabetes has been shown to be clinically effective; however, suboptimal attendance with wide demographic disparities has been reported. To develop quality improvement interventions to maximise attendance, it is important to understand the theoretical determinants (i.e. barriers and enablers) of screening behaviour. The aim of this systematic review is to identify and synthesise the modifiable barriers and enablers associated with diabetic retinopathy screening attendance.

**Methods/design:**

Primary and secondary studies will be included if they report perceived barriers/enablers of diabetic retinopathy screening attendance, from the perspectives of people with diabetes and healthcare providers. There will be no restrictions on study design. Studies will be identified from published and grey literature through multiple sources. Bibliographic databases will be searched using synonyms in four search domains: diabetic retinopathy; screening; barriers/enablers; and theoretical constructs relating to behaviour. Search engines and established databases of grey literature will be searched to identify additional relevant studies. Extracted data will include: participant quotations from qualitative studies, statistical analyses from questionnaire and survey studies, and interpretive descriptions and summaries of results from reports. All extracted data will be coded into domains from the Theoretical Domains Framework (TDF) and (for organisational level data) the Consolidated Framework of Implementation Research (CFIR); with domains representing theoretical barriers/enablers proposed to mediate behaviour change. The potential role of each domain in influencing retinopathy screening attendance will be investigated through thematic analysis of the TDF/ CFIR coding. Domain importance will be identified using pre-specified criteria: “frequency” and “expressed importance”. Variations in perceived barriers and enablers between demographic groups (e.g., socio-economic, ethnic) will be explored.

**Discussion:**

This review will identify important barriers and enablers likely to influence attendance for diabetic retinopathy screening. The results will be used to assess the extent to which existing interventions targeting attendance address the theoretical determinants of attendance behaviour. Findings will inform recommendations for future intervention design.

**Systematic review registration:**

PROSPERO CRD42016032990

**Electronic supplementary material:**

The online version of this article (doi:10.1186/s13643-016-0309-2) contains supplementary material, which is available to authorized users.

## Background

Diabetic retinopathy is the most common microvascular complication of diabetes and one of the leading causes of blindness and visual impairment in people of working age [1, 2]. Although effective treatments are available that can substantially reduce the likelihood of sight threatening complications [3], the success of these treatments is dependent on early detection and timely referral. Systematic surveillance of the diabetic population, with the aim of providing early diagnosis and enabling access to sight-saving treatment, has been shown to be both clinically effective [4] and cost-effective [5].

In some countries, health systems have formal surveillance programmes for diabetic retinopathy detection. For example, in the UK, there is a national population-based diabetic retinopathy screening programme, where people diagnosed with diabetes are systematically invited for screening via a formal registration system [6]. However, globally, less formal surveillance programmes are commonplace, where a more opportunistic screening approach is used [7]. In this protocol, we will be referring to all diabetic retinopathy surveillance programmes as “screening”, as this is the term most frequently adopted in the literature relevant to this review.

Despite potential benefits of screening, attendance is suboptimal, with 20 % of those offered screening in the UK failing to attend and wide variation in screening uptake observed [8]. Screening attendance rates consistently below recommended levels have also been observed internationally (e.g. in the USA), with suboptimal screening attendance shown to be associated with inequalities in outcomes [9–11].

A systematic review of interventions to increase diabetic retinopathy screening attendance identified a number of effective strategies, including those targeting the patient (e.g. increasing patient awareness), the health care practitioner (e.g. improving adherence to recommendations) or the organisation (e.g. improving patient records) [12]. More recently, a systematic review and meta-analysis, assessing the effectiveness of 11 predefined quality improvement (QI) strategies for general diabetes care (e.g. clinician reminders, promotion of self-management, team changes, audit and feedback) found that all QI strategies were associated with increases in diabetic retinopathy screening attendance [13]. However, outcomes across interventions were highly heterogeneous, and it remains unclear which specific QI strategies were most effective and how the intervention strategies might “work”.

The current review is part of a multi-phase programme of work funded by the National Institute of Health Research (NIHR) Health Technology Assessment (HTA), which aims to address these issues (http://www.nets.nihr.ac.uk/__data/assets/pdf_file/0018/158040/PRO-13-137-05.pdf). Phase 1 of this programme of work involves a systematic review and meta-analysis, which has the objective of clarifying what makes one intervention more effective than another. This will be achieved by examining which behaviour change techniques (BCTs), within existing QI interventions, are associated with intervention effectiveness [14]. Phase 2, the current systematic review, sets out to build on this first review and aims to clarify how intervention strategies might work by identifying the modifiable and theoretical determinants of diabetic retinopathy screening attendance.

Theory provides a consistent, generalisable and explanatory framework, alongside an integrated summary of the proposed causal processes involved in behaviour change [15]. Theory enables the potential investigation of how interventions work and the identification of what makes one intervention more effective than another. The benefits of designing behaviour change interventions based on relevant theory are now well recognised [16]. The Medical Research Council guidance for developing and evaluating complex interventions advocates commencing with a “theory phase” in which evidence is accumulated and a theoretical basis for the intervention is developed [17]. There is also evidence that theory-based interventions are often more effective than those that are not [18, 19]. However, the explanatory factors (constructs) from different theories often overlap, making it challenging to identify determinants [20].

The Theoretical Domains Framework (TDF) [21, 22] was developed to address this issue by synthesising 33 theories of behaviour change into 14 “theoretical domains”. Each theoretical domain represents a range of related constructs that may mediate behaviour change at the level of the individual, team or healthcare organisation. For example, the domain “social influences”, includes constructs such as “social support”, “group norms” and “social comparison [20].”

However, it is possible that barriers and enablers could operate at multiple levels in the healthcare system. Ferlie and Shortell [23] have proposed four distinct levels of change that should be considered when designing quality improvement interventions: individual, group or team, overall organisation and wider system or environment. The Consolidated Framework for Implementation Research (CFIR) [24] builds on this and offers a framework of theory-based constructs as a practical guide for systematically assessing potential barriers and facilitators to successful implementation across different organisational levels. This framework includes 39 constructs organised into five domains which are the “intervention characteristics”, “inner setting”, “outer setting”, “characteristics of the individuals involved” and the “process of implementation”. Within the domain of the “inner setting”, for example, there are ten constructs including, but not limited to, the organisation’s “culture”, “structural characteristics” and “incentives and rewards”.

Both the TDF and CFIR have been applied in a number of studies to systematically elicit and characterise barriers and enablers to behaviour change across a range of clinical contexts, primarily through interview and survey studies (see: [20, 25]). Furthermore, the TDF has recently been applied in secondary data analysis as a coding framework for data synthesis as part of three systematic reviews [26–28]. For example, the TDF has been applied as part of a systematic review that aims to identify barriers and enablers to the translation of gestational diabetes guidelines into clinical practice [26]. In this review, Wilkinson et al. used data including routinely collected hospital data, staff surveys, clinic observation and team discussions and evidence from relevant literature. Data were then coded into the TDF domains. The domains found to influence behaviour within this context were: knowledge; beliefs about consequences; intentions; social/professional role/identity; social influences; memory attention and decision processes; and environmental context and resources. Examples of barriers nested within these domains included a lack of staff awareness of gestational diabetes nutrition practice guidelines and clinic dieticians’ and staff’s belief in the importance of dietary modification. Important enablers, identified through clinic observation and team discussions, included a strong clinician-consumer relationship and a positive research audit culture of the organisation.

It is therefore proposed that interventions are more likely to be effective if they influence the causal pathway to screening behaviour by targeting the barriers to screening attendance and attempting to maximise the effect of the enablers. Hence, identifying barriers and enablers in the literature, framing these in terms of theoretical constructs and theoretical domains, and assessing their likely importance for screening attendance, are steps that might explain why some interventions are more effective than others. This would enable intervention designers to optimise interventions by ensuring that they target the likely causal determinants of screening attendance.

## Aim

The current review will adopt a similar approach of applying the TDF/CFIR in a systematic review context to identify the modifiable barriers/enablers to diabetic retinopathy screening from the perspective of people diagnosed with type 1 or type 2 diabetes and their healthcare providers, and frame them in terms of theoretical domains/constructs.

The specific objectives are:

1. To identify the published and grey literature reporting perceived barriers and enablers associated with diabetic retinopathy screening attendance.
2. To extract reported barriers/enablers and categorise these according to TDF/CFIR domains
3. Identify key themes within domains, regarding diabetic retinopathy screening attendance.
4. Apply pre-specified criteria to identify the importance of TDF domains in influencing screening attendance.

## Methods

The protocol has been registered with the PROSPERO International Prospective Register of Systematic Reviews database (reference no: CRD42016032990), and adheres to the Preferred Reporting Items for Systematic Reviews and Meta-Analyses Protocol (PRISMA-P) guidance (see Additional file 1) [29].

### Study eligibility criteria

#### Participants

Anyone diagnosed with type 1 or type 2 diabetes, who are eligible for diabetic retinopathy screening. There will be no restrictions on participants’ demographics or characteristics such as age, gender, ethnicity or location. Additionally, healthcare providers who are responsible for diabetic care will also be included. In the context of this review, healthcare providers are defined as anyone who provides information, guidance, screening or treatment to patients to assist with the management of their diabetes. Such professionals include, but are not limited to: GPs, diabetologists, diabetes specialist nurses, optometrists and ophthalmologists. Populations from any country will be included.

#### Study design

There will be no restrictions on study design. Studies will be included if they (1) investigate or report perceived barriers that might stop a patient diagnosed with diabetes from attending a retinopathy screening appointment and/or (2) investigate or report perceived enablers that might facilitate attendance. Such barriers or enablers could be organisational, emotional, cognitive, behavioural or social; however, they must have the potential to be modifiable, i.e. not demographic or historical factors such as age, gender, ethnicity, socioeconomic status (SES), location or duration of illness.

We will include studies reported in English, conducted within the time period of January 1990 to March 2016, justified on the basis that the St Vincent Declaration [30], which set a target to reduce new blindness in Europe by one third or more, as this is arguably the catalyst for the development of screening programmes for diabetic retinopathy worldwide.

#### Context

There will be no restrictions on population-based retinopathy screening programmes/models (e.g. fixed location screening services, mobile screening services, optometry-based services and mixed services).

#### Data to be extracted

Participants’ (e.g. people with diabetes and healthcare providers) perceptions of diabetic retinopathy screening. Barriers/enablers of interest include factors that participants perceive to influence attendance and are judged by the authors to be modifiable. Extracted data may include, for instance, participant quotations in qualitative studies, quantitative findings from questionnaire and survey studies, alongside interpretive descriptions and summaries of results in published reports.

### Search strategy

#### Identifying published studies

We will search MEDLINE, EMBASE, PsycINFO, Web-of-Science, CENTRAL in the Cochrane Library and Proquest. The search strategy will be adapted to suit each database. Reference lists of any study that meet the inclusion criteria will be screened for any additional studies not identified in the database searches.

We undertook a scoping search to develop an appropriate search strategy and terms were agreed by discussions with the research team. These can be categorised into three distinct concepts: (1) diabetic retinopathy (e.g. diabetic retinopathy; proliferative retinopathy; diabetic eye disease); (2) screening (e.g. screening; vision tests; ophthalmoscopy; eye examination; fundus photography); and (3) potential barriers and enablers (e.g. (non)compliance, (non)responsive), including terms relating to background characteristics of the population where there is evidence in the literature of disparities in health screening attendance (e.g. patient acceptance of healthcare; health promotion; healthcare disparities; socioeconomic factors; education; and ethnic groups).

In addition to the terms listed above, the search strategy includes terms related to each of the TDF domains, to ensure the potential range of barriers and enablers are represented. These were agreed by a consensus group exercise. Consensus group participants (seven research psychologists who were familiar with the TDF and CFIR) were presented with the TDF domain labels and component constructs and asked to rank the constructs in terms of the extent to which they represent/summarise the domain. The highest ranking (i.e., the top two constructs) within each domain were selected and added to the list of key words representing the TDF. For example, in the domain “emotions” the top two constructs selected were “anxiety” and “fear”. An example for the search strategy is provided (see Additional file 2).

#### Identifying grey literature

This will include an internet search using the search engine Google and established sources of grey literature [e.g. conference abstracts, OpenGrey, PsycEXTRA, The Healthcare Management Information Consortium (HMIC) database] using the search terms: “diabetic retinopathy” AND screening AND attendance AND [barrier* OR “facilitate* OR enable].

We will consult with the project representative stakeholder group to identify further sources of grey literature. The group includes experts in diabetes care, representatives of the UK screening programme, patients, practitioners, professional organisations and policy makers.

### Study selection process

Following de-duplication the lead review author (EGR) will screen the titles and abstracts of all the references to decide whether the full text manuscript should be retrieved. Each study will be judge as either (a) not meeting the “eligibility criteria” or (b) potentially meeting the “eligibility criteria” for inclusion. To estimate screening reliability, a second review author (FL) will independently screen the titles and abstract of 20 % of the references generated against eligibility criteria.

**Fig. 1:**
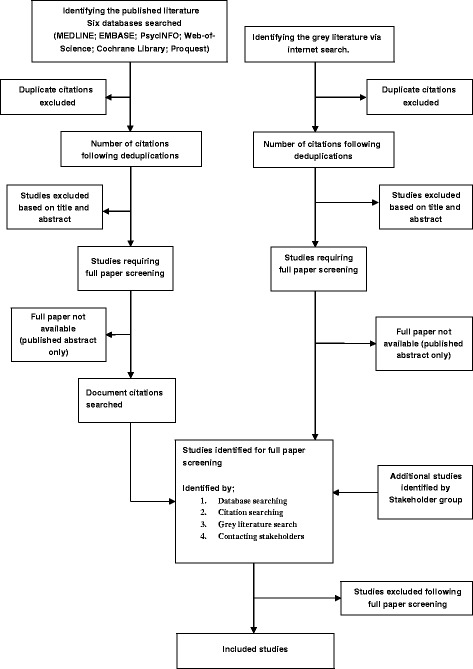
Flow diagram of the proposed article screening process

The full-text manuscript of potentially eligible studies will then be assessed for inclusion against the “eligibility criteria” by the lead review author (EGR). A second review author (FL) will independently screen 20 % for inclusion. Any discrepancies during study selection between the two review authors will be resolved by consensus, or discussed with a third review author (JF) (see Fig. 1: PRISMA diagram).

### Quality assessment

For study description purposes, we will assess the quality of the literature using appropriate tools. We will use the Authority, Accuracy, Coverage, Objectivity, Date, and Significance (AACODS) checklist (https://dspace.flinders.edu.au/jspui/bitstream/2328/3326/4/AACODS_Checklist.pdf) for the grey literature; instruments recommended by the Cochrane Qualitative Research Methods Group e.g. the Critical Appraisal Skills Programme (CASP) (http://media.wix.com/ugd/dded87_29c5b002d99342f788c6ac670e49f274.pdf) for qualitative studies; and the Effective Public Health Project tool for published quantitative studies [31].

The lead review author (EGR) will quality-assess all studies and a second review author (FL) will assess a random sample of 20 % of studies. Any differences of opinion regarding quality will be resolved by consensus, or discussed with a third author (JF).

### Data extraction and analysis materials/tools

We will develop a data extraction form to extract study characteristics, including: country, setting (e.g. home, hospital), design, participants, data analysis methods and main findings.

We will prepare a coding manual with the definitions for each of the 14 theoretical domains from the TDF, and the 39 distinct constructs from the CFIR, to facilitate coding consistency and reliability.

### Analysis

**Fig. 2:**
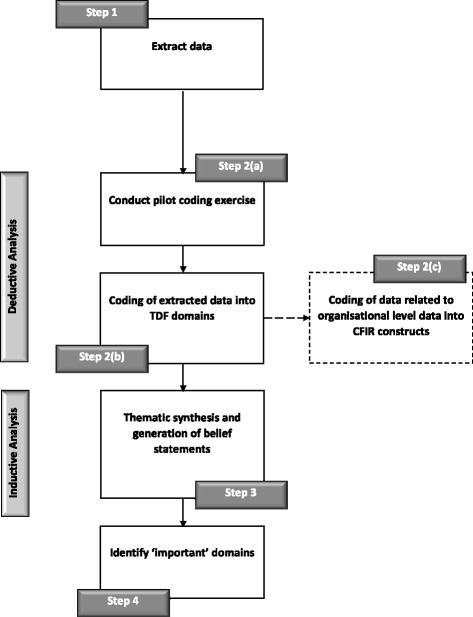
Flow diagram of steps in the proposed analysis

To the extent that is possible in a secondary analysis context, we will follow established analysis methods that have been used in previous studies applying the TDF to interview transcripts from semi-structured interviews (e.g. [32–34]). These methods typically follow a combined content and framework analysis approach, involving the following steps outline below (see Fig. 2 for a flow diagram).

Step 1: Data extraction

The first review author will identify and extract data reporting participants’ perceptions of diabetic retinopathy screening attendance from each included study. Extracted data will be tabulated in an excel spreadsheet (one spreadsheet per study). Each data point will be categorised as either (1) raw data (e.g. participant quotations from qualitative studies); (2) analysed data from result sections (e.g. thematic analysis, and statistical analyses); and (3) interpretative descriptions and summaries of results from published reports. A second review author will extract and categorise data reporting participants’ perceptions of screening attendance from 20 % of included studies, and inter-rater reliability for data extraction assessed using Cohen’s Kappa. A minimum kappa value of 0.75 will be taken to represent high agreement [35].

#### Deductive analysis

Step 2: Coding of extracted data

2a.Pilot coding exercise

In order to develop coding heuristics to facilitate data analysis and promote greater consistencies in coding, three studies will be randomly selected to practise applying the TDF to code extracted data (step 1) into theoretical domains [36]. The lead review author (EGR) will jointly code these three studies alongside a second review author (FL). Any disagreement or uncertainty will be resolved through consensus or discussed with a third review author (JF).

2b.TDF coding

The lead review author (EGR) will go on to code the data extracted from all remaining included studies. Using the TDF as a coding framework, extracted data will be coded according to which TDF domain they are judged to represent. For example, an extracted quote from a patient saying, “I believe that having my eyes screened once a year will help preserve my vision”, would be coded in the domain “Beliefs about consequences”. If a reported barrier/enabler was judged to concurrently represent more than one domain, it will be coded into multiple domains. For example, if a patient is quoted as saying “I am anxious about attending my screening appointment because I can’t drive home after receiving eye drops”, this would be coded to both the “emotions” and “beliefs about capabilities” domain.

To assess coding reliability the second review author (FL) will code extracted data into TDF domains for 20 % of included studies, and inter-coder reliability assessed as per step 1.

2c.CFIR coding

To ensure that we adequately consider the potential organisational factors that might influence screening attendance, we will re-examine data coded into TDF domains that relate to the organisational level (e.g. “environmental context and resources”) using the CFIR. We will consider all the CFIR domains and constructs. This additional step will help elaborate aspects relating to organisational level barriers/enablers.

#### Inductive analysis

Step 3: Thematic analysis and generation of belief statements

In line with a framework analysis approach, step 3 will focus on sifting and sorting the data to thematically synthesise and identify key emerging issues [37, 38]. In a consensus group exercise between the review authors (EGR, FL, JJF), we will group together similar extracted data that have been coded into each TDF domain/CFIR construct that express similar views relating to perceived barriers/enablers to diabetic retinopathy screening attendance. We will then generate, by consensus, a summary belief label for each cluster of similar grouped data summarising these shared views.

#### Importance criterion

Step 4: Assess the importance of TDF domains (and when appropriate CFIR constructs)

Each domain identified in step 2 will be reviewed against two established “importance criteria” [39], to determine which domains are likely to be the most important for influencing screening attendance. First, we will assess “frequency”, by identifying (a) the level of elaboration of each domain in terms of the number of beliefs within each domain and (b) the number of studies that identify each belief within each domain. Second, we will consider “expressed importance” within each domain, by looking for a statement from the authors’ interpretation of the study findings articulating that beliefs were reported to be important by the study participants. Although this process is less precise than consideration of frequencies, and will require discussion and judgement by the research team, it has good fit with the qualitative approach by considering the meaning, interpretation and prioritisation of the data by authors who have closer familiarity with the primary data than will be possible for the review team. We will interpret the domains that have the highest combined “frequency” and “expressed importance” as the most important in regard to diabetic retinopathy screening attendance.

If there are sufficient data, we will explore whether the domains identified as “important” vary according to patients’ demographic variables such as age, type of diabetes (e.g. type 1 vs type 2), and duration of diabetes, socio-economic status and ethnicity. If there are sufficient data to compare views across the different healthcare professions involved in diabetic care, these will also be explored.

## Discussion

This review employs a systematic and replicable approach towards identifying barriers and enablers associated with diabetic retinopathy screening attendance from the perspective of both people with diabetes and healthcare providers. Through the explicit use of theoretical constructs, this review will be able to conceptualise the potential determinants of screening behaviour. The combination of deductive coding (informed by conceptual frameworks to guide barrier identification) and inductive analysis (to allow unanticipated findings and patient insights to emerge) is a strength of this approach.

A potential key limitation to this review is that we are relying on reported and interpreted data. Therefore, there is a potential for a “reporting bias” as the studies may present selective findings to fit the stated research question and might not fully report all the findings and data that are relevant to this enquiry. It is also possible that the results will be poorly described in these studies as inadequate reporting is a frequent reported limitation of study manuscripts in both the implementation and behaviour change literature [40, 41]. However, we reason that relying on the existing primary and secondary literature is the most systematic and replicable approach to identifying barriers and enablers across a variety of screening programmes, demographic backgrounds, research groups and research perspectives.

### Implications for research

This review will provide an evidence-base from which to evaluate existing interventions aimed at increasing diabetic retinopathy screening attendance. Specifically, phase 3 of the multi-phase programme of work will assess the extent to which QI interventions, designed to maximise retinopathy screening attendance, target the theoretical domains that are important in determining attendance. This will be achieved by mapping the BCTs identified in the phase 1 review against the theoretical domains identified in phase 2 (the current review), using an established mapping matrix [42]. The findings can help to establish a set of recommendations of which theoretical domain(s) to target and which to avoid, thus enhancing the likely effectiveness of diabetic retinopathy screening programmes.

## Abbreviations

AACODS, Authority, Accuracy, Coverage, Objectivity, Date, and Significance; BCT, behaviour change techniques; CASP, Critical Appraisal Skills Programme; CFIR, Consolidated Framework of Implementation Research; HMIC, The Healthcare Management Information Consortium; HTA, Health Technology AssessmentNIHR National Institute of Health Research; PRISMA-P, Preferred Reporting Items for Systematic Reviews and Meta-Analyses Protocol; QI, quality improvement; TDF, Theoretical Domains Framework

## Additional files

Additional file 1: Prisma-P Checklist. This checklist includes recommended items to address in a systematic reviews protocol and their location in this protocol. (PDF 60 kb) Additional file 2: Search strategy for MEDLINE. Specific search strategy used for the database MEDLINE comprising of both keywords and Mesh terms. (PDF 26 kb)
