# Supplementary material for: Barriers and enablers to diabetic retinopathy screening attendance: Protocol for a systematic review
Source: Syst Rev. 2016 Aug 11;5:134. doi: 10.1186/s13643-016-0309-2 (PMC4981960; doi:10.1186/s13643-016-0309-2)
Supplement: Additional file 1: — Prisma-P Checklist. This checklist includes recommended items to address in a systematic reviews protocol and their location in this protocol. (PDF 60 kb) [file 13643_2016_309_MOESM1_ESM.pdf]

Additional file 1: Prisma-P Checklist

Section/Topic	Item	Checklist Item	Page
Administrative Information			
Title			
Identification	1a	Identify the report as a protocol of a systematic review	P.1
Update	1b	If the protocol is for an update of a previous systematic review, identify as such	N/A
Registration	2	If registered, provide the name of the registry (e.g. PROSPERO) and registration number	P.3/8
Authors			
Contact	3a	Provide name, institutional affiliation, and e-mail address of all protocol authors; provide physical mailing address of corresponding author	P.1
Contributions	3b	Describe contributions of protocol authors and identify the guarantor of the review	25
Amendments	4	If the protocol represents an amendment of a previously completed or published protocol, identify as such and list changes; otherwise, state plan for documenting important protocol amendments	N//A
Support			
Sources	5a	Indicate sources of financial or other support for review	P24
Sponsor	5b	Provide name for the review funder and/or sponsor	P24
Role of sponsor/funder	5c	Describe role of funder(s), sponsor(s), and/or institution(s), if any, in developing the protocol	P24
INTRODUCTION			
Rationale	6	Describe the rationale for the review in the context of what is already known	P7/8
Objectives	7	Provide an explicate statement of the question(s) the review will address with reference to participants, interventions, comparators and outcomes (PICO)	P8
METHODS			
Eligibility criteria	8	Specify the study characteristics (e.g. PICO, study design, setting, time frame) and report characteristics (e.g., years considered, language, publication status) to be used as criteria for eligibility for the review	P8-9
Information sources	9	Describe all intended information source (e.g., electronic databases, contact with study authors, trial registers, or other grey literature sources) with planned dates of coverage	P10-11

Search strategy	10	Present draft of search strategy to be used for at least one electronic database, including planned limits, such that it could be repeated	P30-33
Study records			
Data management	11a	Describe the mechanism(s) that will be used to manage records and data throughout the review	P.12
Selection process	11b	State the process that will be used for selecting studies (e.g., two independent reviewers) through each phase of the review (i.e., screening, eligibility, and inclusion in meta-analysis	P.11/12
Data collection process	11c	Describe planned methods of extracting data from reports (e.g., piloting forms, done independently, in duplicate), any processes of obtaining and confirming data from investigators	P 12
Data items	12	List and define all variables for which data will be sort (e.g., PICO items, funding sources), any pre-planned data assumptions and simplifications	NA
Outcomes and prioritisation	13	List and define all outcomes for which data will be sort, including prioritisation or main and additional outcomes, and rationale	NA
Risk of bias in individual studies	14	Describe anticipated methods for assessing risk of bias of individual studies, including whether this will be done at the outcome or study level, or both; state how this information will be used in data synthesis	P.12
DATA			
Synthesis	15a	Describe criteria under which data will be quantitatively synthesised	N/A
	15b	If data are appropriate for quantitative synthesis, describe planned summary measures, methods of handling data, and methods of combining data from studies, including any planned exploration of consistency	N/A
	15c	Describe any proposed additional analyses (e.g., sensitivity, or subgroup analysis, meta-regression.	P16
	15d	If quantitative synthesis is not appropriate, describe the type of summary planned	P14-16
Meta-bias(es)	16	Specify any planned assessment of meta-bias(es) (e.g., publication bias across studies, selective reporting within studies)	N/A
Confidence in cumulative evidence	17	Describe how the strength of the body of evidence will be assessed (e.g. GRADE)	N/A
