# Supplementary material for: Barriers and enablers to diabetic retinopathy screening attendance: Protocol for a systematic review
Source: Syst Rev. 2016 Aug 11;5:134. doi: 10.1186/s13643-016-0309-2 (PMC4981960; doi:10.1186/s13643-016-0309-2)
Supplement: Additional file 2: — Search strategy for MEDLINE. Specific search strategy used for the database MEDLINE comprising of both keywords and Mesh terms. (PDF 26 kb) [file 13643_2016_309_MOESM2_ESM.pdf]

Additional file 2: Search strategy for MEDLINE

1 exp Diabetic Retinopathy/

2 ((diabet$ or proliferative or non-proliferative) adj4 retinopath$).tw.

3 diabetic retinopathy.kw.

4 (diabet$ adj3 (eye$ or vision or visual$ or sight$)).tw.

5 (retinopath$ adj3 (eye$ or vision or visual$ or sight$)).tw.

6 (DR adj3 (eye$ or vision or visual$ or sight$)).tw.

7 or/1-6)

8 exp Mass Screening/

9 exp Vision Tests/

10 exp Telemedicine/

11 exp Photography/

12 exp Ophthalmoscopes/

13 exp Ophthalmoscopy/

14 (ophthalmoscop$ or fundoscop$ or funduscop$).ti.

15 ((exam$ or photo$ or imag$) adj3 fundus).tw.

16 (photography or retinography).tw.

17 ((mydriatic or digital or retina$ or fundus or steroscopic) adj3 camera).tw.

18 ((mydriatic or digital or retina$ or fundus or steroscopic) adj3 imag$).tw.

19 screen$.tw.

20 ((eye$ or retina$ or ophthalm$) adj4 exam$).tw.

21 ((eye or vision or retinopathy or ophthalmic) adj4 test$).tw.

22 ((eye$ or retina$ or ophthalm$) adj4 visit$).tw.

23 Office Visits/

24 (telemedicine$ or telemonitor$ or telescreen$ or telehealth or teleophthalmology).tw.

25 or/8-24

26 exp Patient Acceptance of health Care/

27 exp Attitude to Health/

28 exp Health Behavior/

29 Motivation/

30 Fear/

31 exp Self Concept/

32 Personal Autonomy/

33 Self Care/

34 Behavior Therapy/

35 (barrier$ or obstacle$ or facilitat$ or enable$).tw.

36 (knowledge or skill$ or role$ or identity or capabilit$ or optimis$ or consequence$ or reinforcement or intention$ or goal$ or memory or attention or context$ or resources or emotion$).tw.

37 (decision adj2 process$).tw.

38 (social adj2 influence$).tw.

39 (behavioural adj2 regulation).tw.

40 (competence or self-efficac$ or self-confidence or incentiv$ or reward$ or anxiety fear$ or self-monitor$ or habits).tw.

41 (outcome adj2 expectanc$).tw.

42 (action adj2 plan$).tw.

43 (decision adj2 mak$).tw.

44 (social adj2 (support$ or norm)).tw.

45 ((behaviour$ or behavior$) adj3 (change$ or modif$ or activat$ or control$ or amend$)).tw.

46 (uptake or takeup or attend$ or accept$ or adhere$ or attitude$ or participat$ or facilitat$ or utilisat$ or utilizat$).tw.

47 (motivat$ or satisf$ or promot$ or consent$ or self select$ or self referr$).tw.

48 (complie$ or comply or compliance$ or noncompliance$ or non compliance$).tw.

49 (encourag$ or discourage$ or reluctan$ or nonrespon$ or non respon$ or refuse$).tw.

50 (non-attend$ or non attend$ or dropout or drop out or apath$).tw.

51 Health Education/

52 exp Patient Education as Topic/

53 exp Health Promotion/

54 exp Counseling/

55 "Attitude of Health Personnel"/

56 (health adj2 (promotion$ or knowledge or belief$)).tw.

57 (educat$ adj2 (intervention$ or information or material or leaflet)).tw.

58 Focus groups/

59 Interviews as Topic/

60 (focus adj3 group$).tw.

61 Socioeconomic Factors/

62 exp Poverty/

63 Social Class/

64 Educational Status/

65 ((school or education$) adj3 (status or level$ or attain$ or achieve$)).tw.

66 Employment/

67 Uncompensated Care/

68 Reimbursement Mechanisms/

69 Reimbursement, Incentive/

70 (insurance adj3 (health$ or scheme$)).tw.

71 (financial or pay or payment or copayment or paid or fee or fees or monetary or incentiv$ or disincentiv$).tw.

72 Healthcare Disparities/

73 Health Status Disparities/

74 exp Medically Underserved Area/

75 Rural Population/

76 Urban Population/

77 exp Ethnic Groups/

78 Minority Groups/

79 Vulnerable Populations/

80 ((health$ or social$ or racial$ or ethnic$) adj5 (inequalit$ or inequit$ or disparit$ or equit$ or disadvantage$ or depriv$)).tw.

81 (disadvant$ or marginali$ or underserved or under served or impoverish$ or minorit$ or racial$ or ethnic$).tw.

82 or/26-80

83 7 and 25 and 82)

84 limit 83 to yr="1990 -Current"

85 (ranibizumab or bevacizumab or avastin or aflibercept).ti.

86 (cataract$ or intraocular or glaucoma$ or phaco$ or photocoagulat$ or photodynamic or laser$ or vitrectom$).ti.

87 (macula$ adj2 (degener$ or oedema or edema)).ti.

88 nerve fiber layer.ti.

89 (coronary or cardiac or cardio$ or heart or myocardia$ or artery or aneurysm or atrial or echocardiography or hypertension or hypotension or stroke or pulmonary or COPD or lung$ or organ$ or smoking).ti.

90 (pregnan$ or gestational or neonat$ or perinatal or maternal or trimester or congenital or ovary or breast$).ti.

91 (kidney or liver or cirrhosis or renal or hepatitis or dialysis or pancrea$ or gastric or gastrectom$ or surg$ or duoden$).ti.

92 (blood glucose or blood pressure or ketoacidosis or hypoglycemi$ or rosiglitazone).ti.

93 (lipid$ or lipase$ or statin$ or hypercholesterolemia or microalbumin$ or albumin$ or platlet$ or plasma$ or hemoglobin$ or haemochromat$ or arterial).ti.

94 (cancer$ or carcinoma$ or neoplas$ or adenoma$ or metformin$).ti.

95 (urin$ or incontinence or bladder or constipat$ or bowel$ or faecal or colorectal or colon$).ti.

96 (gene$ or genotype$ or genome$ or genomic or phenotyp$ or biomarker$ or polymorphism$ or interleukin$).ti.

97 (cell$ or molecular or assay).ti.

98 (cystic or fibrosis or CF or tuberculosis or TB or lupus).ti.

99 (neuropath$ or nephropath$ or prematurity).ti.

100 ($arthritis or steroid$ or osteoporosis or atherosclerosis or sclerosis).ti.

101 (apnea or sleep or limb or oral$ or celiac or coeliac or skin or MRSA or anesthesia or vitamin or HIV or testosterone or erectile or schizophren$ or bipolar or antipsychotic$ or psychotic$).ti.

102 prevalence.ti.

103 or/85-102

104 84 not 103

***************************
